# Antiseizure medications for idiopathic generalized epilepsies: a systematic review and network meta-analysis

**DOI:** 10.1007/s00415-023-11834-8

**Published:** 2023-06-28

**Authors:** Hongyuan Chu, Xinyu Zhang, Jie Shi, Zhirui Zhou, Xu Yang

**Affiliations:** 1 Department of Neurology, Peking University Aerospace School of Clinical Medicine (Aerospace Center Hospital), No. 15, Yuquan Road, Haidian District, Beijing, 100049 China; 2 Radiation Oncology Center, Huashan Hospital, Shanghai Medical College, Fudan University, Shanghai, 200040 China; 3 Department of Pediatrics, Peking University First Hospital, Beijing, China; 4 Department of Neurology, Peking University First Hospital, Beijing, China; 5 Department of Neurology, Tsinghua University Yuquan Hospital, Beijing, 100040 China

**Keywords:** Antiseizure medications, Idiopathic generalized epilepsy, Absence, Myoclonic, Tonic–Clonic

## Abstract

**Objectives:**

To compare the efficacy and safety of antiseizure medications (ASMs), both as monotherapies and adjunctive therapies, for idiopathic generalized epilepsies (IGEs) and related entities.

**Methods:**

Two reviewers independently searched PubMed, Embase, and the Cochrane Library for relevant randomized controlled trials from December 2022 to February 2023. Studies on the efficacy and safety of ASM monotherapies or adjunctive therapies for IGEs and related entities—including juvenile myoclonic epilepsy, childhood absence epilepsy (CAE), juvenile absence epilepsy, or generalized tonic–clonic seizures alone (GTCA)—were included. Efficacy outcomes were the proportions of patients remaining seizure free for 1, 3, 6, and 12 months; safety outcomes were the proportions of any treatment-emergent adverse event (TEAE) and TEAEs leading to discontinuation. Network meta-analyses were performed in a random-effects model to obtain odds ratios and 95% confidence intervals. Rankings of ASMs were based on the surface under the cumulative ranking curve (SUCRA). This study is registered with PROSPERO (No. CRD42022372358).

**Results:**

Twenty-eight randomized controlled trials containing 4282 patients were included. As monotherapies, all ASMs were more effective than placebo, and valproate and ethosuximide were significantly better than lamotrigine. According to the SUCRA for efficacy, ethosuximide ranked first for CAE, whereas valproate ranked first for other types of IGEs. As adjunctive therapies, topiramate ranked best for GTCA as well as overall for IGEs, while levetiracetam ranked best for myoclonic seizures. For safety, perampanel ranked best (measured by any TEAE).

**Conclusions:**

All of the studied ASMs were more effective than placebo. Valproate monotherapy ranked best overall for IGEs, whereas ethosuximide ranked best for CAE. Adjunctive topiramate and levetiracetam were most effective for GTCA and myoclonic seizures, respectively. Furthermore, perampanel had the best tolerability.

**Supplementary Information:**

The online version contains supplementary material available at 10.1007/s00415-023-11834-8.

## Introduction

Historically, idiopathic generalized epilepsies (IGEs) have included juvenile myoclonic epilepsy (JME), childhood absence epilepsy (CAE), juvenile absence epilepsy (JAE), and generalized tonic–clonic seizures alone (GTCA) [1]. The term “idiopathic” refers to “self-originating” or “spontaneously arising” and implies that the condition is genetic [2]. Characterized by 2.5–6 Hz generalized spike waves, IGEs account for approximately 15–20% of all epilepsies, as well as 55% of newly diagnosed generalized epilepsy in children and adolescents [3, 4]. Because IGEs have a strong underlying genetic basis, the updated classification from the International League Against Epilepsy suggested that IGEs should be a subgroup of “genetic generalized epilepsies,” but reserved the term to describe the aforementioned four overlapping syndromes [1].

The diagnosis of IGEs has important implications for their treatment and prognosis. In patients with IGEs, development, neurological examinations, and radiographic results are typically normal [5]. Additionally, because most cases arise in children and adolescents, IGEs are often emphasized to be of pediatric importance only; however, considerable psychosocial symptoms—such as mood disorders, attention deficits, and learning disabilities—can be observed until adulthood [6, 7]. Long-term follow-up studies have revealed correlations between IGEs and outcomes such as poorer employment/financial conditions, decreased interactions with families, and unplanned pregnancies [8]. Thus, although IGEs may seem easier to manage than symptomatic or partial epilepsies, they should not receive less attention than these other epilepsies.

Antiseizure medications (ASMs) are the cornerstone of treatment for IGE syndromes. A good response rate, at 60–80% of seizure control (i.e., more than 1 year without seizure), can be achieved with appropriate ASM selection [9, 10]. First-line monotherapy controls symptoms in the majority of patients with IGEs. Among the first-line treatments, valproate (rather than lamotrigine or topiramate) monotherapy is the recommended first choice for IGEs in boys and men because it was shown to have better efficacy and tolerability in the SANAD study (Level I evidence) [11]. By contrast, levetiracetam monotherapy is favored in women able to have children; it seldom induces drug–drug interactions. Although levetiracetam was inferior to valproate in the SANAD II study [12], it has shown good efficacy in seizure control in cohort studies [13]. The choice of optimal initial monotherapy in IGEs is very important. Management decisions are different for JME, CAE, JAE, and CTSA, and need to be individualized. However, a limited number of randomized controlled trials (RCTs) have compared various ASMs head-to-head as initial monotherapies for IGEs and related subsyndromes. For example, some ASMs, such as carbamazepine or oxcarbazepine, may exacerbate absence seizures, whereas lamotrigine and gabapentin can exacerbate some myoclonic seizures [14]. A comprehensive integration of the evidence is thus needed so that a tailored plan can be developed for each patient.

Adjunctive therapy should be started when two different monotherapies have been unable to successfully control IGEs. The drug of choice generally depends on the main seizure subtype. Lamotrigine and levetiracetam are recommended as adjunctive therapies to valproate, except in JME. Topiramate, zonisamide, and perampanel have also demonstrated efficacy in RCTs or observational studies and are recommended adjunctive options (e.g., in myoclonic seizures). However, the majority of adjunctive medications do not have proof of efficacy in placebo-controlled RCTs. Hence, a comparison of the efficacy and tolerability of adjunctive ASMs remains lacking; clinically, the choice of adjunctive drug often relies on class III or IV evidence. Furthermore, with numerous established and new medications currently available, physicians face difficult decisions when choosing the most appropriate adjunctive drugs because of the limited high-quality evidence [15].

To the best of our knowledge, no previous review has compared the efficacy and tolerability of ASMs for IGEs (neither as monotherapies nor as adjunctive therapies). This network meta-analysis (NMA) aims to provide comprehensive evidence for the relative efficacy and safety of ASMs for controlling IGEs.

## Methods

This NMA was conducted following a protocol that was prospectively registered with PROSPERO (No. CRD42022372358) and adhered to the Preferred Reporting Items for Systematic Review and Meta-Analysis (PRISMA) statement for network meta-analysis [16].

### Search strategy

Reviewers searched PubMed, Embase, and the Cochrane Library for relevant RCTs. Search terms were limited to the type of epilepsy, antiseizure medication, type of study, and year of publication (Appendix Table S1); there were no limitations on language. The reference lists of relevant RCTs and reviews were searched manually. The search procedure was conducted from December 2022 to February 2023, and EndNote X9 was used for reference management.

### Study selection

In the first stage of review, two authors independently selected studies by screening the titles, abstracts, and content according to the inclusion and exclusion criteria. Differences in opinion were discussed to obtain consensus, as necessary; disagreements were arbitrated by the senior reviewer.

#### Inclusion criteria

(1) Population: patients of any age or sex who were diagnosed with IGEs, JME, CAE, JAE, or GTCA. (2) Intervention: monotherapy or adjunctive therapy with ASMs. (3) Outcomes: efficacy outcomes (the proportion of participants with seizure reduction or freedom after 1, 3, 6, or 12 months) and safety outcomes (the proportion of patients who experienced any treatment-emergent adverse event [TEAE], or serious TEAEs leading to discontinuation).

#### Exclusion criteria

(1) Patients with a diagnosis of another type of epilepsy. (2) Animal or cellular research. (3) Observational study or review article. (4) Incomplete outcomes with no explanation of clinical relevance.

### Data extraction and quality evaluation

Data extraction was collected on standardized spreadsheets and double-checked. If multiple articles reported outcomes from the same population, the most comprehensive outcome was noted. When studies reported different terms of follow-up, all non-overlapping information was included. Version 2 of the Cochrane Collaboration Risk of Bias tool (RoB2) for assessing randomized trials was used to evaluate the included studies [17].

### Statistical analysis

Statistical analysis was primarily conducted using R software (version 4.2.1, http://www.r-project.org). The gemtc package was used in JAGS 4.3.0 for the analysis (https://CRAN.R-project.org/package=R2jags). Pairwise meta-analyses were performed using a random-effects model for outcomes of the included studies to obtain odds ratios (ORs) and 95% confidence intervals (CIs). The NMA was conducted within a Bayesian framework that assumed a binomial likelihood for the number of events per medication [18, 19]. For outcomes with two or more treatment arms, the arms were pooled to form a single node for the corresponding ASM. The Markov chain Monte Carlo method was used to compare multiple ASMs by synthesizing the results of direct and indirect comparisons [20]. Each model used four Markov chains; the initial interaction value was set to 5000 and the adjusted interaction number was 10,000. The *I*^2^ statistic was calculated to quantify heterogeneity; *I*^2^ > 50% was defined as high-grade heterogeneity [21]. The local inconsistency model was assessed using a node-splitting method in which significance was set at a two-tailed *p* value of 0.05. The surface under the cumulative ranking (SUCRA) curves and the mean ranks were used to evaluate different ASMs, with a higher SUCRA representing superior efficacy.

## Results

### Identification and description of studies

**Fig. 1:**
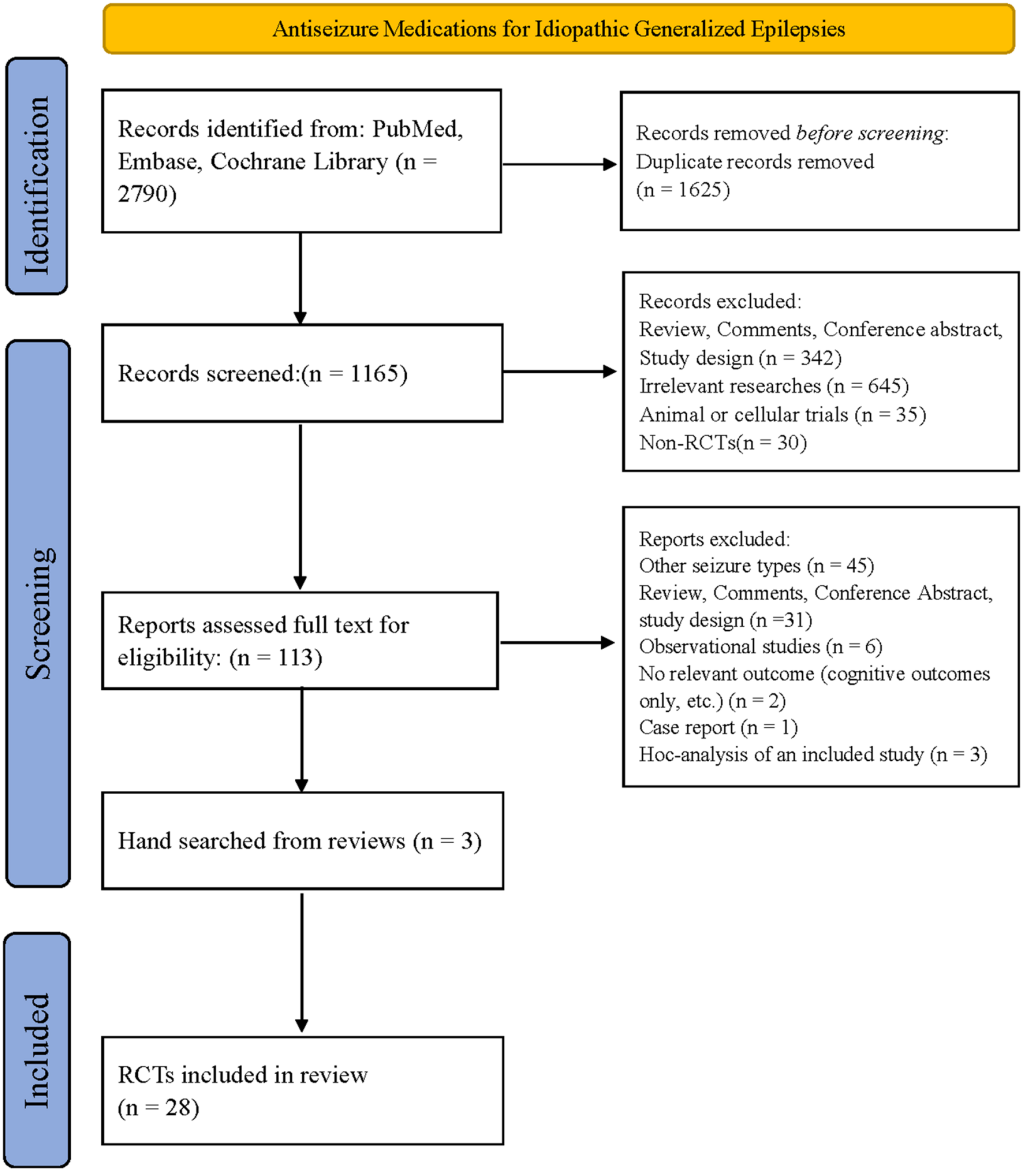
Flow chart of data retrieval

Of the 2790 abstracts that were identified from PubMed, Embase, and the Cochrane Library, 113 were assessed for eligibility by full-text review, of which 87 were excluded. Finally, 28 RCTs containing 4282 patients were included in the NMA (Fig. 1).

The demographic characteristics of patients, such as sex and age of entrance and onset, were noted. Detailed information is provided in Table 1. All 28 RCTs assessed the efficacy and safety of ASMs in IGEs or the related entities CAE, JAE, JME, or GTCA (arm of: valproate *n* = 14, lamotrigine *n* = 13, levetiracetam *n* = 6, perampanel *n* = 5, topiramate *n* = 3, ethosuximide *n* = 5, lacosamide *n* = 1). The number of patients assigned to each ASM, the initial and maximum dose of daily use, and the time of follow-up are shown in Table 2. Study designs—including region, blinding, conflict of interest, and register—were carefully checked to ensure the reliability of results (Table 3). Furthermore, RoB2 assessments were conducted to evaluate bias (Appendix Table S2).

**Table 1:** Characteristics of the Included Studies

	Participant information									
Study ID	Number Randomized	Seizure type description	Mean age		Newly diagnosed	Refractory	Male		Age at onset	
			Arm1	Arm2			Arm1	Arm2	Arm1	Arm2
AE										
Basu 2005 [22]	30	Typical AE	Range 5–14		No	No	16		/	/
Callaghan 1982 [23]	28	Typical AE	8.00	9.00	No	No	8	5	Range 2–5	Range 3–6
Cnaan 2017 [24]	208	CAE	7.60		No	Yes	84		/	/
Coppola 2004 [25]	38	CAE or JAE	7.50		Yes	No	17		7.5	
Fattore 2011 [26]	59	CAE or JAE	Range 4–15		Yes	No	15	12	/	/
Frank 1999 [27]	29	Typical AE	Range 2–16		Yes	No	5	5		
Glauser 2010, 2013 [28, 29]	453	CAE	Range 2.5–13		Yes	No		195		
Huang 2009 [30]	45	CAE	7.00	6.00	Yes	No	10	16	8-month	7-month
Shinnar 2017 [6]	382	CAE	7.60		Yes	No	43%		/	/
Hwang 2011 [31]	128	AE	Range 4–10		Yes	No	19	20	6.3	6.4
Brandt 2020 [32]	60	AE and ME	28.50	27.40	No	No	18	16	/	/
ME										
Machado 2013 [33]	72	JME	26.80	27.30	No	No	15	10	16.3	15.3
Nejad 2009 [34]	42	JME	/	/	No	No	/	/	/	/
Noachtar 2008 [35]	120	iIGEs with myoclonic seizures	25.00	26.80	No	No	22	22	11.9	14.1
Levisohn 2007 [36]	28	JME	15.00	16.00	No	No	6	5	Range 8–26	
Brandt 2020 [37]	47	AE and ME	28.50	27.40	No	No	18	16	/	/
IGEs										
Berkovic 2007 [38]	164	GTCA associated with IGEs	26.90	30.60	No	No	34	39	11.6	
SANAD arm B 2007 [11]	716	Mainly IGEs, some unclassified	22.80	22.50	No	No	142	143	/	/
SANAD arm B 2007	716	Mainly IGEs, some unclassified	22.30	/	No	No	142		/	/
SANAD II arm B 2021 [39]	520	Mainly IGEs, some unclassified	14.10	13.60	Yes	No	170	167	13	12
GTCA										
Driscoll 2020 [40]	144	Primary GTCA	25.40	26.20	No	No	33	32	11.6	10.3
French 2015 [41]	162	idiopathic GTCA	27.30	29.50	No	Yes	35	36	11.7	10.9
French 2020 [42]	138	GTCA	26.60	29.10	No	Yes	29	30	11.8	10.3
Prakash 2016 [43]	60	idiopathic GTCA	31.70	33.60	Yes	No	20	17	31	33
Vossler 2020 [44]	242	GTCA	27.80	27.60	No	Yes	55	45	12.9	12.9
Wu 2018 [45]	251	GTCA	31.50	32.80	No	No	79	76	14.5	16.5
Biton 2010 [46]	117	Primary GTCA	26.90	24.90	No	Yes	21	18	11.9	12.1
Beran 1998 [47]	26	AE, ME, and GTCA	29.00		No	Yes	15		7.4	
Biton 1999 [48]	80	Primary GTCA	26.80	25.60	No	No	24	21	26	25

*AE* absence epilepsy, *CAE* childhood absence epilepsy, *JAE* juvenile absence epilepsy, *ME* myoclonic epilepsy, *JME* juvenile myoclonic epilepsy, *IGEs* idiopathic generalized epilepsies, *GTCA* generalized tonic–clonic seizures alone

“/” represents not mentioned or not taken down for reasons

**Table 2:** Description of Intervention in the included studies

Intervention information								
Study ID	Monotherapy	Arm		Initial daily dose		Maximum daily dose		Follow-up
		Arm 1 (number)	Arm 2 (number)	Arm1	Arm 2	Arm1	Arm 2	
AE								
Basu 2005	Yes	VPA 15	LTG 15	/	/	/	10 mg/kg/day	12 months
Callaghan 1982	Yes	ESM 14	VPA 14	250 mg/d	1500 mg/d	400 mg/d	2400 mg/d	18 months-4 years
Cnaan 2017 (arm 1 2)	Yes	ESM 75	LTG 55	/	/	2000 mg/d	600 mg/d	12 months
Cnaan 2017 (arm 3)		VPA 78		/		3000 mg/d		
Coppola 2004	Yes	LTG19	VPA19	0.5 mg/kg/d	10 mg/kg/d	12 mg/kg/d	30 mg/kg/d	12 months
Fattore 2011	Yes	LEV38	Placebo21	10 mg/kg/day	/	30 mg/kg/day	/	602 days
Frank 1999	Yes	LTG15	Placebo14	0.5 mg/kg/day		15 mg/kg/day		25 weeks
Glauser 2010, 2013 (arm 1 2)	Yes	ESM156	LTG 149	10 mg/kg/d	0.3 mg/kg/d	60 mg/kg/d	12 mg/kg/d	12 months
Glauser 2010, 2013 (arm 3)		VPA148		10 mg/kg/d		60 mg/kg/d		12 months
Huang 2009	Yes	VPA23	LTG 22	15 mg/kg/d	0.15 mg/kg/d	30 mg/kg/d	10 mg/kg/d	12 months
Shinnar 2017 (arm 1 2)	Yes	ESM91	LTG 91	10 mg/kg/d	0.3 mg/kg/d	60 mg/kg/d	12 mg/kg/d	12 months
Shinnar 2017 (arm 3)		VPA96		10 mg/kg/d		60 mg/kg/d		
Hwang 2011	Yes	ESM48	VPA59	10 mg/kg/d	10 mg/kg/d	23 mg/kg/d	26 mg/kg/d	3.4 years(1-17 years)
Brandt 2020	no	PER 51	Placebo56	2 mg/day	/	8 mg/day	/	136 weeks
ME								
Machado 2013	yes	LTG41	VPA31	25 mg/d	200 mg/d	300 mg/d	3000 mg/d	24 months
Nejad 2009	yes	LTG22	VPA20	500 mg/d	200 mg/d	1500-2000 mg/d	800 mg/d	28 weeks
Noachtar 2008	No	LEV60	Placebo62	1000 mg/d	/	3000 mg/d	/	30 weeks
Levisohn 2007	Yes	TPM19	VPA9	3–4 mg/kg/day	10 mg/kg/day	9 mg/kg/day	60 mg/kg/day	26 weeks
Brandt 2020	No	PER 24	Placebo 23	2 mg/day	/	8 mg/day	/	136 weeks
IGEs								
Berkovic 2007	Yes	LEV80	Placebo84	Adults: 1000 mg/day children:20 mg/kg/day	/	Adults: 3000 mg/day children: 60 mg/kg/day	/	20 weeks
SANAD arm B 2007 (arm 1 2)	Yes	LTG 239	VPA 238	Decided by clinician				83.5 months
SANAD arm B 2007 (arm 3)	Yes	TPM 239		Decided by clinician				83.5 months
SANAD II arm B 2021	Yes	LEV260	VPA260	250 mg/d	500 mg/d	500 mg/d	500 mg/d	67.9 months
GTCA								
Driscoll 2020	No	Adjunctive PER 72	Placebo 72	10 mg/kg/day	/	10 mg/kg/day	/	12 weeks
French 2015	No	Adjunctive PER 81	Placebo81	2 mg/day	/	8 mg/day	/	21 weeks
French 2020	No	Adjunctive PER 68	Placebo70	2 mg/day	/	8 mg/day	/	2 years
Prakash 2016	Yes	VPA30	LTG30	10 mg/kg/day	0.5 mg/kg/day	30 mg/kg/day	12 mg/kg/day	12 months
Vossler 2020	No	Adjunctive LCM 121	Placebo121	100 mg/day	/	300–400 mg/day	/	28 weeks
Wu 2018	No	Adjunctive LEV126	Placebo125	1000 mg/day	/	3000 mg/day	/	36 weeks
Biton 2010	No	Adjunctive LTG58	Placebo59	12.5-50 mg/day	/	150-500 mg/day	/	24 weeks
Beran 1998	No	Adjunctive LTG26	Placebo26	50 mg/day	/	150 mg/day	/	24 weeks
Biton 1999	No	Adjunctive TPM39	Placebo41	50 mg/day	/	400 mg/day	/	20 weeks

*AE* absence epilepsy, *ME* myoclonic epilepsy, *IGEs* idiopathic generalized epilepsies, *GTCA* generalized tonic–clonic seizures alone, *VPA* valproate, *LTG* lamotrigine, *TPM* topiramate, *LEV* levetiracetam, *ESM* ethosuximide, *PER* perampanel, *LCM* lacosamide

“/” represents not mentioned or not taken down for reasons

**Table 3:** Design information of included studies

Study Design Information						
Study ID	Region	Multicenter	Double-blind	Conflict of interest/disclosure	Children/elderly/pregnancy	Register
AE						
Basu 2005	India	No	Not stated	Not stated	Children	Not stated
Callaghan 1982	Eire	No	Yes	Not stated	Children	Not stated
Cnaan 2017	The USA	32 sites	Open-label	None	Children	NCT00088452
Coppola 2004	Italy	No	Open-label	Not stated	Children	Not stated
Fattore 2011	Italy	11center	Open-label	Consultancy fees and/or research grants from the manufacturers of ASMs disclosed	Children	EudraCT 2005–003520-18,2005–003520-26
Frank 1999	The USA	Unclear	Yes	Stated	Children	Protocol 105–044
Glauser 2010&2013	The USA	32 sites	Open-label	Study medications were provided free of charge by Pfizer, Abbott Laboratories, and GlaxoSmithKline	Children	NCT00088452
Huang 2009	China	No	Open-label	Not stated	Children	/
Shinnar 2017	The USA	32 sites	Open-label	Stated	Children	NCT00088452
Hwang 2011	Japan	No	Open-label	Not stated	Children	Not stated
ME						
Machado 2013	Cuba	Tertiary center	No	Stated	Children	/
Nejad 2009	Iran	No	Open-label	Not stated	No	/
Noachtar 2008	14 countries	37 centers	Yes	Study medications were provided free of charge by Pfizer, Janssen-Cilag, Desitin, Eisai, and Sanofi-Synthelab	No	NCT00150774-N166
Levisohn 2007	The USA	No	Open-label	Not stated	No	Not stated
IGEs						
SANAD arm B 2007	The UK	Yes	No	Stated	No	SP0993
SANAD II arm B 2021	The UK	Yes	No	Stated	No	ISRCTN30294119
Berkovic 2007	Europe, North America, Mexico, Australia, and New Zealand	50 centers	Yes	Grants from manufacturers of ASMs disclosed	/	NCT00150748
Brandt 2020	In 16 countries	78 sites	Yes	Funded by Eisai Inc	/	NCT01393743
GTCA						
Driscoll 2020	21 countries	Yes	Yes	Several authors are full-time employees of Pfizer	Children and adults	NCT01747915
French 2015	In 16 countries	78 sites	Yes	Funded by Eisai Inc	/	NCT01393743
French 2020	In 16 countries	78 sites	Yes	Funded by Eisai Inc	/	NCT01393743
Prakash 2016	India	No	No	Not stated	/	/
Vossler 2020	North America, Latin America, Europe and the Asia–Pacific region	Yes	Yes	Speaker honoraria from Eisai, Greenwich Biosciences, Lundbeck, Sunovion and UCB Pharma etc	/	SP0982, NCT02408523
Wu 2018	China and Japan	115sites	Yes	Sponsored by UCB Pharma	/	N01159; NCT01228747)
Biton 2010	International (not detailed)	38 sites	Yes	Funded by GlaxoSmithKline, manufacturer of lamotrigine	/	Glaxo-SmithKline protocol LAM40097
Beran 1998	Australia	5sites	No	Not stated	/	/
Biton 1999	The United States and Costa Rica	8 sites	Yes	Not stated	/	/

*AE* absence epilepsy, *ME* myoclonic epilepsy, *IGEs* idiopathic generalized epilepsies, *GTCA* generalized tonic–clonic seizures alone

“/” represents not mentioned or not taken down for reasons

A favorable consistency of the included studies was identified using the node-splitting method (all *p* $>$ 0.05). Moreover, heterogeneity was low in the included studies (all *I*^2^ $<$ 27%).

### Efficacy outcomes

**Fig. 2:**
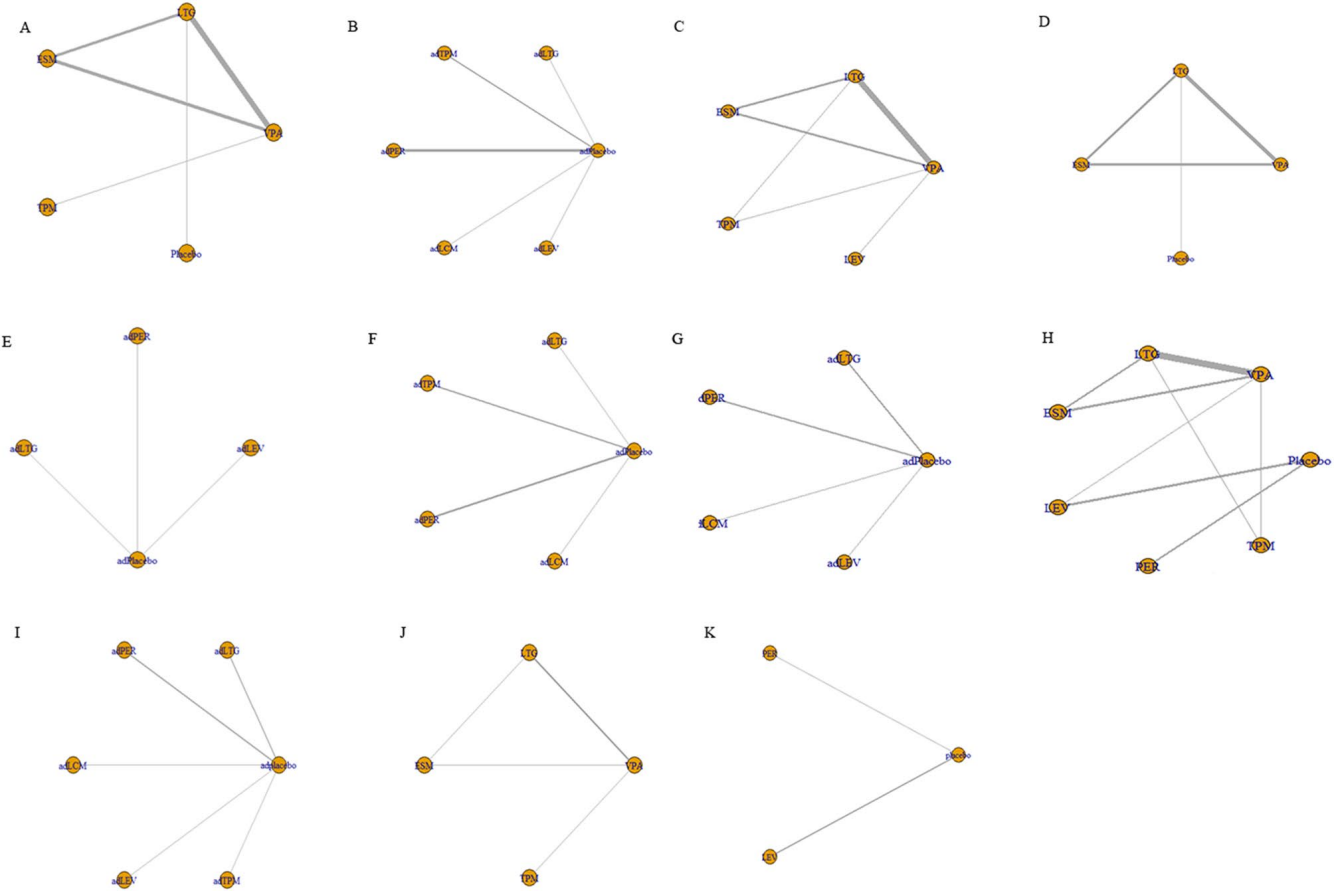
Network of treatment comparisons for efficacy and safety. **A** Seizure free for 3–6 months after monotherapy in overall idiopathic generalized epilepsies (IGEs). **B** Seizure free for 3–6 months after adjunctive therapy in overall IGEs. **C** Seizure free for 12 months after monotherapy in overall IGEs (long-term follow-up). **D** Seizure free for 3–6 months after monotherapy in absence epilepsies (AE). **E** Seizure free for 3–6 months after adjunctive therapy in myoclonic epilepsies (ME). **F** Seizure free for 3–6 months after adjunctive therapy in generalized tonic–clonic seizures alone (GTCA). **G** Any adverse event after adjunctive therapy in overall IGEs. **H** Any adverse event after monotherapy in overall IGEs. **I** Adverse events leading to discontinuation after adjunctive therapy in overall IGEs. **J** Adverse events leading to discontinuation after monotherapy in overall IGEs (part 1). **K** Adverse events leading to discontinuation after monotherapy in overall IGEs (part 2). *AE* absence epilepsy, *ME* myoclonic epilepsy, *IGEs* idiopathic generalized epilepsies, *GTCA* generalized tonic–clonic seizures alone, *VPA* valproate, *LTG* lamotrigine, *TPM* topiramate, *LEV* levetiracetam, *ESM* ethosuximide, *PER* perampanel, *LCM* lacosamide, *ad* adjunctive

The included RCTs provided outcomes regarding the proportion of patients who achieved seizure freedom for 1, 3, 6, and 12 months after ASM treatment. The majority of studies reported the intention-to-treat population; intention-to-treat outcomes were thus analyzed rather than per-protocol outcomes. The network-evidence map plots of seizure-free outcomes for ASMs as monotherapies and adjunctive therapies are shown in Fig. 2A–F. Both short-term seizure-free outcomes (3–6 months) and relatively long-term outcomes (12 months) were analyzed.

**Fig. 3:**
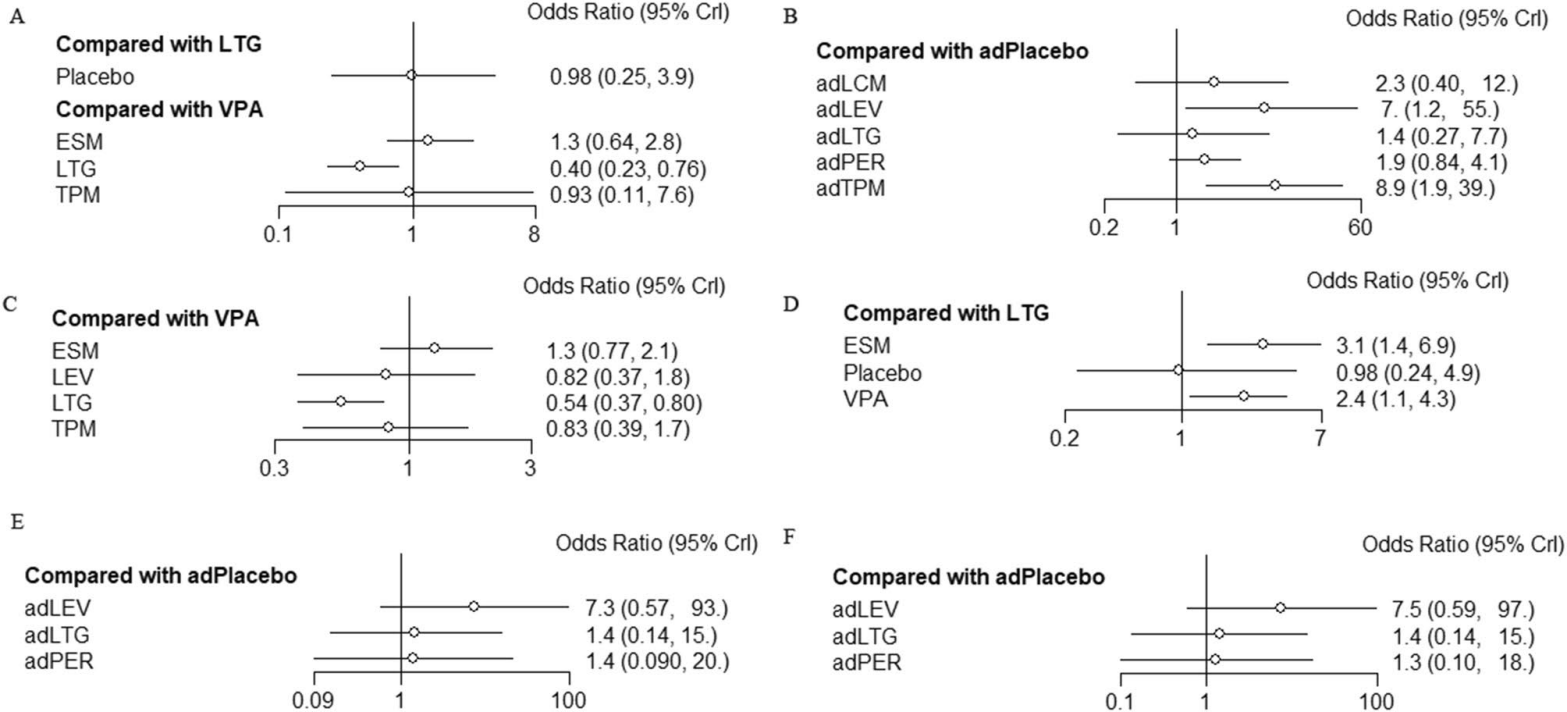
Forest plots of treatment comparisons for efficacy. **A** Seizure free for 3–6 months after monotherapy in overall idiopathic generalized epilepsies (IGEs). **B** Seizure free for 3–6 months after adjunctive therapy in overall IGEs. **C** Seizure free for 12 months after monotherapy in overall IGEs (long-term follow-up). **D** Seizure free for 3–6 months after monotherapy in absence epilepsies (AE). **E** Seizure free for 3–6 months after adjunctive therapy in myoclonic epilepsies (ME). **F** Seizure free for 3–6 months after adjunctive therapy in generalized tonic–clonic seizures alone (GTCA). *AE* absence epilepsy, *ME* myoclonic epilepsy, *IGEs* idiopathic generalized epilepsies, *GTCA* generalized tonic–clonic seizures alone, *VPA* valproate, *LTG* lamotrigine, *TPM* topiramate, *LEV* levetiracetam, *ESM* ethosuximide, *PER* perampanel, *LCM* lacosamide, *ad* adjunctive

The forest plot of the NMA revealed that all ASMs were associated with a higher rate of either short- or long-term seizure-free outcomes compared with placebo (Fig. 3). In the monotherapy analysis for overall IGEs, ethosuximide had a higher 3- to 6-month seizure-free rate than valproate (OR = 1.3, 95% CI = 0.66–2.8), whereas lamotrigine had a significantly lower rate than valproate (OR = 0.40, 95% CI = 0.23–0.77; Fig. 3A).

In the adjunctive therapy analysis, all ASMs showed superior efficacy to placebo; the effects of levetiracetam (OR = 7, 95% CI = 0.07–14) and topiramate (OR = 8.9, 95% CI = 1.9–39) were significant (Fig. 3B). There were no significant differences in long-term (12-month) follow-up outcomes between adjunctive valproate and adjunctive ethosuximide (OR = 1.3, 95% CI = 0.77–2.1), levetiracetam (OR = 0.82, 95% CI = 0.37–1.8), or topiramate (OR = 0.83, 95% CI = 0.39–1.7); however, adjunctive lamotrigine had significantly lower efficacy than adjunctive valproate (OR = 0.54, 95% CI = 0.37–0.8; Fig. 3C).

Subsyndromes of IGEs were also independently analyzed. In absence epilepsies, ethosuximide (OR = 3.1, 95% CI = 1.4–6.9) and valproate (OR = 2.4, 95% CI = 1.1–4.3) had significantly superior efficacy to lamotrigine as monotherapies (Fig. 3D). However, in the analysis of adjunctive therapies in myoclonic epilepsies (Fig. 3E) and GTCA (Fig. 3F), there were no significant differences between ASMs and placebo, likely because the 95% CIs were very broad.

### Safety outcomes

**Fig. 4:**
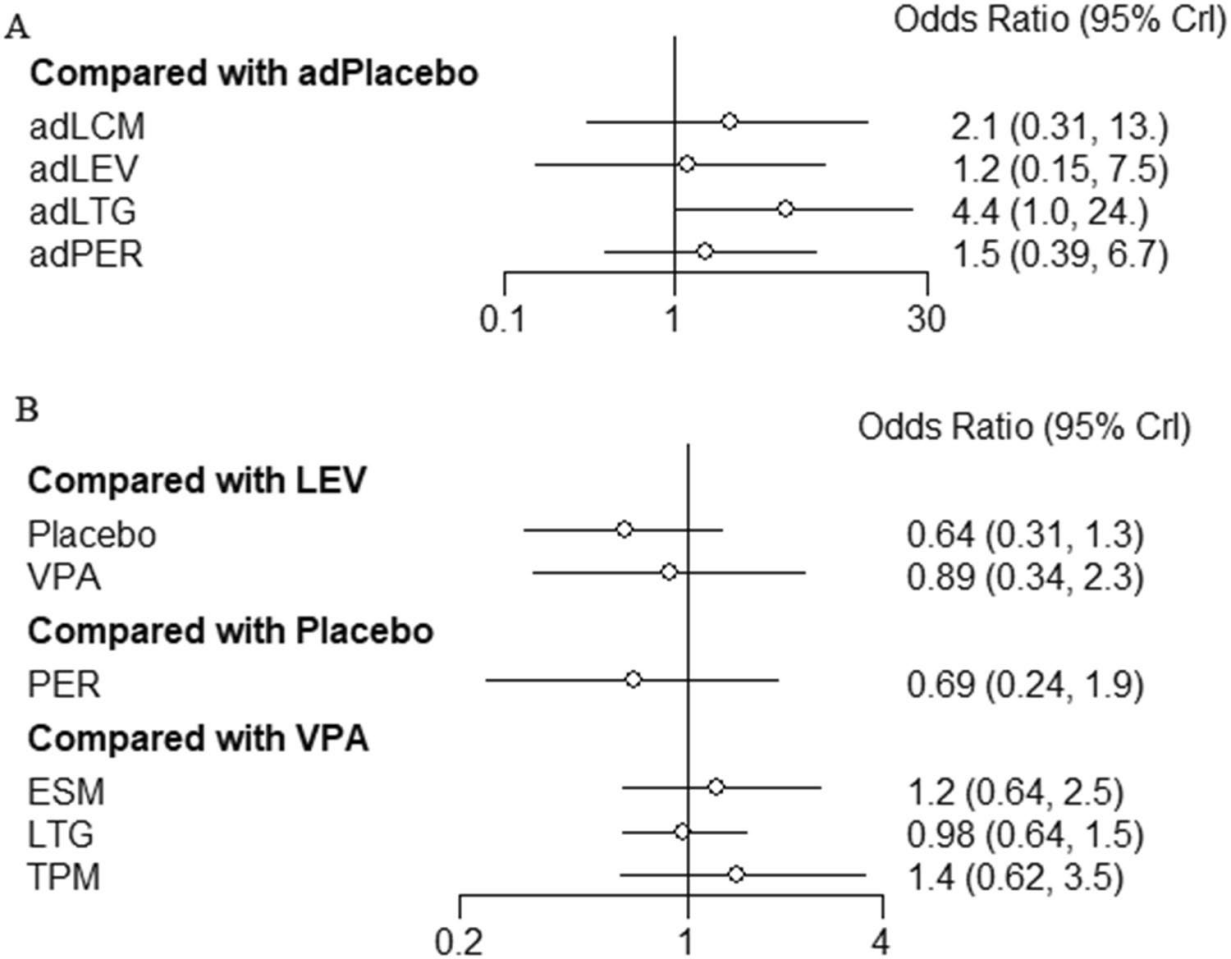
Forest plots of treatment comparisons for safety. **A** Any adverse event after adjunctive therapy in overall idiopathic generalized epilepsies (IGEs). **B** Any adverse event after monotherapy in overall IGEs. *IGEs* idiopathic generalized epilepsies, *VPA* valproate, *LTG* lamotrigine, *TPM* topiramate, *LEV* levetiracetam, *ESM* ethosuximide, *PER* perampanel, *LCM* lacosamide, *ad* adjunctive

In overall IGEs, adjunctive lamotrigine (OR 4.4, 95% CI = 1.0–24) had a significantly increased risk of any TEAEs compared with adjunctive placebo (Fig. 4A). There were no significant differences in safety between ASMs as either adjunctive therapies (Fig. 4A) or monotherapies (Fig. 4B).

### SUCRA

**Fig. 5:**
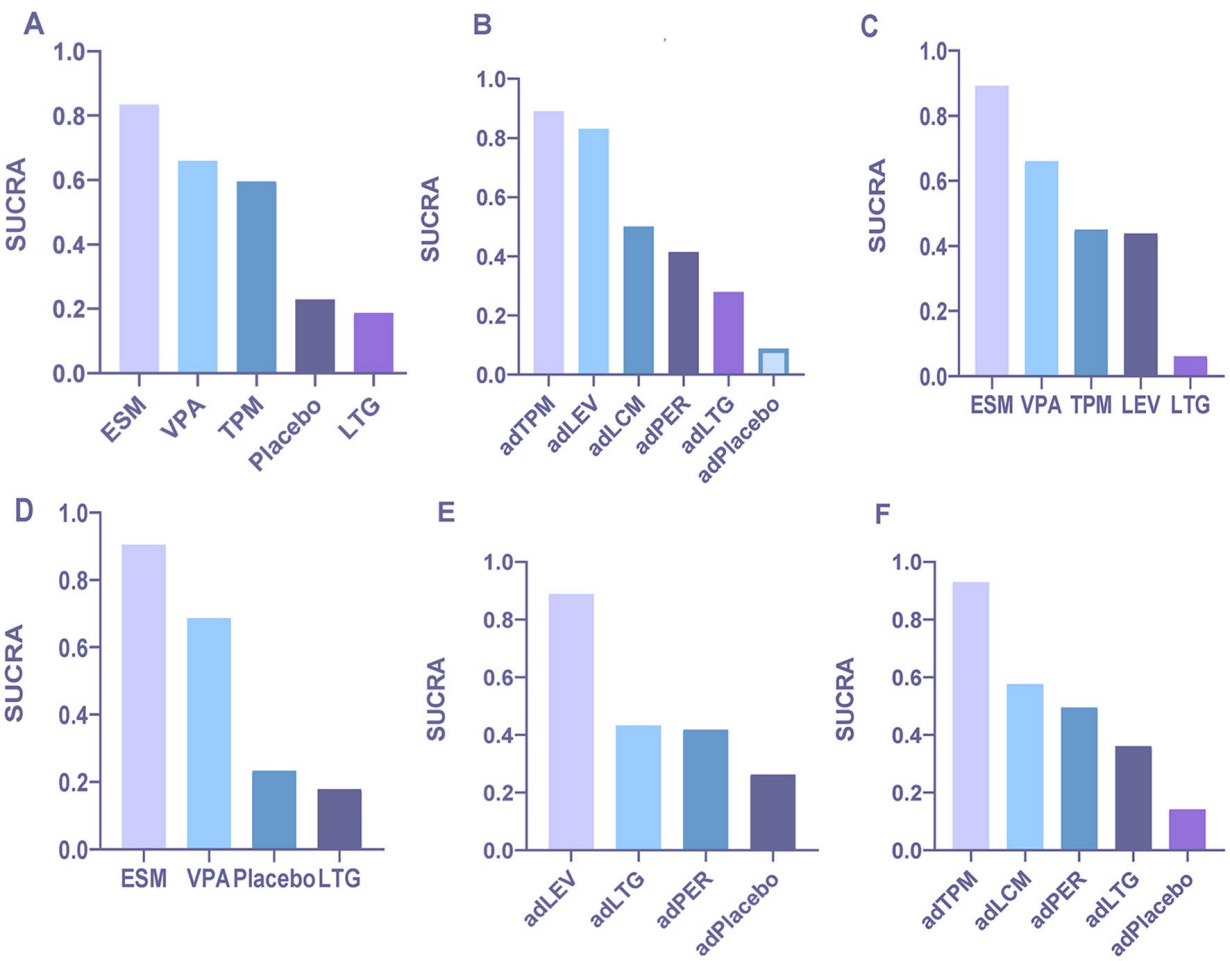
Ranking of efficacy outcomes according to the surface under the cumulative ranking curve (SUCRA). **A** Seizure free for 3–6 months after monotherapy in overall idiopathic generalized epilepsies (IGEs). **B** Seizure free for 3–6 months after adjunctive therapy in overall IGEs. **C** Seizure free for 12 months after monotherapy in overall IGEs (long-term follow-up). **D** Seizure free for 3–6 months after monotherapy in absence epilepsies (AE). **E** Seizure free for 3–6 months after adjunctive therapy in myoclonic epilepsies (ME). **F** Seizure free for 3–6 months after adjunctive therapy in generalized tonic–clonic seizures alone (GTCA). *AE* absence epilepsy, *ME* myoclonic epilepsy, *IGEs* idiopathic generalized epilepsies, *GTCA* generalized tonic–clonic seizures alone, *VPA* valproate, *LTG* lamotrigine, *TPM* topiramate, *LEV* levetiracetam, *ESM* ethosuximide, *PER* perampanel, *LCM* lacosamide, *ad* adjunctive

According to the SUCRA, the efficacy ranking for monotherapies was ethosuximide > valproate > topiramate > placebo > lamotrigine in overall IGEs, and the efficacy ranking for adjunctive therapies was topiramate > levetiracetam > lacosamide > perampanel > lamotrigine > placebo. For 12-month seizure-free efficacy, the ranking was ethosuximide > valproate > topiramate > levetiracetam > lamotrigine (Fig. 5A–C and Appendix Table S2A–C). In absence epilepsies, the SUCRA efficacy ranking for monotherapies was ethosuximide > valproate > placebo > lamotrigine. In myoclonic seizures, the efficacy ranking for adjunctive therapies was levetiracetam > lamotrigine > perampanel > placebo. Moreover, for GTCA, the efficacy ranking for adjunctive therapies was topiramate > lacosamide > perampanel > lamotrigine > placebo (Fig. 5D–F and Appendix Table S3D–F).

**Fig. 6:**
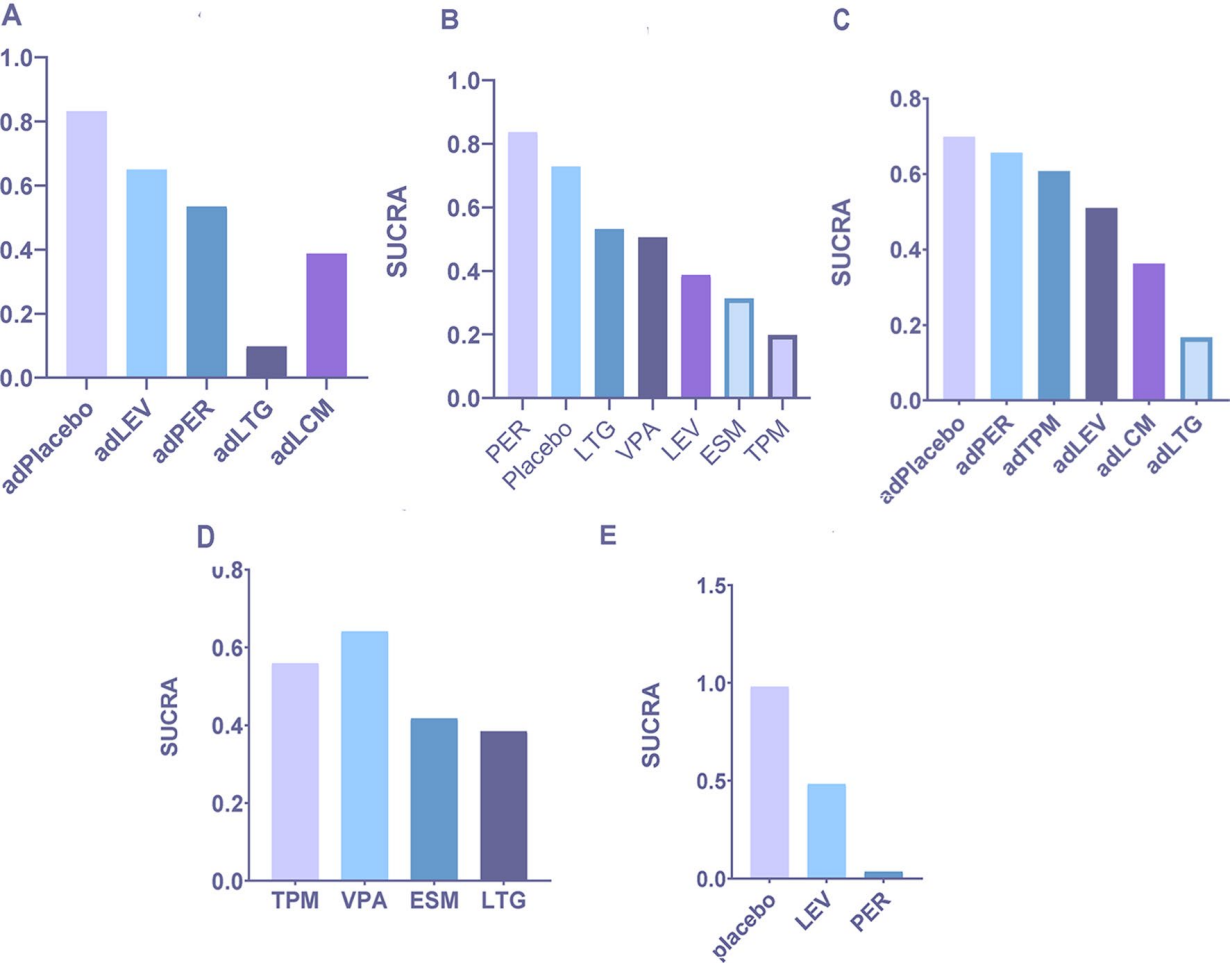
Ranking of safety outcomes according to the surface under the cumulative ranking curve (SUCRA). **A** Any adverse event after adjunctive therapy in overall idiopathic generalized epilepsies (IGEs). **B** Any adverse event after monotherapy in overall IGEs. **C** Adverse event leading to discontinuation after adjunctive therapy in overall IGEs. **D** Adverse event leading to discontinuation after monotherapy in overall IGEs (part 1). **E** Adverse event leading to discontinuation after monotherapy in overall IGEs (part 2). *AE* absence epilepsy, *ME* myoclonic epilepsy, *IGEs* idiopathic generalized epilepsies, *GTCA* generalized tonic–clonic seizures alone, *VPA* valproate, *LTG* lamotrigine, *TPM* topiramate, *LEV* levetiracetam, *ESM* ethosuximide, *PER* perampanel, *LCM* lacosamide, *ad* adjunctive

In overall IGEs, the SUCRA ranking of associations with more total TEAEs for adjunctive therapies was placebo > levetiracetam > perampanel > lamotrigine > lacosamide; for monotherapies, it was perampanel > placebo > lamotrigine > valproate > levetiracetam > ethosuximide (Fig. 6A, B and Appendix Table S3G-H). For serious TEAEs leading to discontinuation, the safety ranking was placebo > perampanel > topiramate > levetiracetam > lacosamide > lamotrigine for adjunctive therapy (Fig. 6C and Appendix Table S3I) and topiramate > valproate > ethosuximide > lamotrigine; placebo > levetiracetam > perampanel for monotherapy (Fig. 6D–E and Appendix Table S3J–K).

## Discussion

Our NMA indicated that all of the included ASMs were more effective than the placebo. The network forest plots compared monotherapies with valproate and adjunctive therapies with placebo. Significant superiority was identified for adjunctive levetiracetam and topiramate, while inferiority was identified for lamotrigine monotherapy. Non-significant differences were also identified. Using SUCRA, rankings of efficacy and tolerability were summarized.

The analyses of the efficacy outcomes of being seizure free for 3–6 or 12 months did not affect the status of valproate as the first-choice monotherapy for overall IGEs without contraindications. Although ethosuximide ranked first according to SUCRA, its optimal efficacy and tolerability are probably only favorable for absence epilepsies rather than for overall IGEs, and especially tonic–clonic seizures [49]. It is supported as the drug of choice for absence seizures without other seizure types, in accordance with other reviews and guidelines (April 2022, NICE guidelines, https://www.nice.org.uk/guidance/ng217).

Lamotrigine monotherapy unexpectedly had the lowest efficacy in both short- and long-term seizure-free outcomes in the SUCRA analysis and showed significant inferiority in the forest plots for overall IGEs. Although a longer duration of titration may partly account for this short-term result (because lamotrigine must be titrated very slowly to avoid unwanted side effects), this finding is in accordance with a SANAD study suggesting that lamotrigine should not be interpreted as a “broad spectrum” antiseizure medication because it provides worse seizure control than valproate or topiramate in generalized epilepsies [11]. However, these findings should be interpreted with caution. Adjunctive lamotrigine is advantageous in controlling unclassified generalized tonic–clonic seizures (i.e., those unable to be classified as IGEs or partial epilepsy) [50].

In recent years, levetiracetam has been increasingly prescribed and recommended. The present NMA supports its efficacy as a second-line monotherapy and as an ideal adjunctive choice in overall IGEs according to its efficacy ranking. Although it was not found to be a non-inferior monotherapy to valproate in a previous study [12], its favorable efficacy, fast action, and good tolerability (leading to less TEAEs than placebo in our ranking) indicate its considerable potential. However, longitudinal studies are needed in the future (for both levetiracetam and brivaracetam).

In the present study, adjunctive topiramate ranked first in adjunctive therapies according to SUCRA. As a second-generation ASM, topiramate is especially effective in JME and GTCA [51]. In a Cochrane review, the efficacy of topiramate monotherapy in JME was not significantly different from that of valproate (the current drug of choice) [51]. Although topiramate is associated with cognitive TEAEs such as dulling or memory problems [52], which are especially unfavorable in neurodevelopmental disorders, its tolerability was the best ranked in terms of TEAEs leading to discontinuation.

The head-to-head comparison of third-generation ASMs used as adjunctive therapies is of great importance because there is a lack of accumulated evidence, especially for perampanel and lacosamide. In the present analysis, SUCRA demonstrated that their efficacies seem to fall between those of levetiracetam and lamotrigine. In addition, lacosamide may be more effective than perampanel for seizure-free outcomes in GTCA. Although there was previously a lack of high-quality long-term evidence, recent unblinded controlled studies have revealed that perampanel reduces generalized seizures with a median of 90% in 52-week follow-up, and has the potential to increase seizure freedom [42, 53, 54]. Moreover, after 24 weeks of lacosamide adjunctive treatment, the freedom rate from generalized seizures was 27.5% (versus placebo 13.2%) in an RCT [44].

In the current study, perampanel was the best-ranked therapy for tolerability as both a monotherapy and an adjunctive therapy when any adverse event was considered. The characteristic TEAEs of perampanel are irritability and aggression [52, 55]. In contrast, when ranking the therapies in terms of serious TEAEs leading to discontinuation, perampanel was inferior to placebo and levetiracetam. Similarly, the PERMIT study indicated a discontinuation rate of 17.6% at 12 months, in which psychiatric TEAEs were the most common reason for discontinuation [56]. We thus predict a future in which new-generation ASMs, like perampanel, are used to control generalized seizures. However, more high-quality research is warranted to draw stronger conclusions.

We must note that, although all included ASMs significantly improved the seizure-free rate compared with placebo, ASMs can neither cure epilepsies nor treat the underlying pathology that causes them; they merely aim to stop the occurrence of seizure symptoms. A better understanding of the molecular mechanisms underlying the pathogenesis, epileptogenesis, and pharmacoresistance of epilepsies is needed to change our clinical approach. For example, genetic therapies and stem cell therapies will likely cure epilepsies in the future [57].

Despite this, the importance of ASMs should not be ignored, although many patients do not achieve or retain complete seizure freedom. Improved seizure reduction may significantly downgrade the risk of injury and unexpected death [58]; however, in the present review, the NMA of the seizure reduction rate had to be stopped because insufficient data were provided in the included studies. In addition, although some well-controlled complex epilepsies might be disrupted by a single poor night’s sleep or missing dose and breakthrough, substantial improvements have been achieved [59]. Compared with invasive options like vagus nerve stimulation or corpus callosotomies, the use of established and new ASMs may provide more tolerable, incremental benefits. Furthermore, the increase in available ASMs makes it possible to devise more individualized plans, thus benefitting patients. Longitudinal comprehensive studies are therefore warranted to evaluate efficacy in particular populations (or genotypes), more in-detail tolerability, effects on quality of life, and cost-utility for ASMs.

The present study had some limitations. Methodologically, a limited number of outcomes restrained us from analyzing other important efficacy outcomes, such as seizure reduction or electroencephalogram improvements. Furthermore, because specific TEAEs were not evaluated, the tolerability outcome analysis lacked details, and only rough results were obtained because of a lack of information. The search strategy mainly focused on the idiopathic generalized seizure type. Thus, some important ASMs (such as cenobamate, brivaracetam, etc.) most frequently used in focal epilepsies, although recently proven adjunctive use in generalized seizures, were not involved. More meaningful future studies are necessary to elucidate the efficacy of these ASMs.

Although low heterogeneity was identified according to the *I*^2^ test, differences between RCTs existed such as the inclusion criteria, time of treatment, and concomitant drugs. Furthermore, although a statistically suitable and well-known analysis was used, certain overestimations or underestimations may still exist. For example, relatively broad 95% CIs were obtained because relatively few RCTs were included. Further studies are therefore required to further confirm our conclusions.

## Conclusions

Among the included ASM monotherapies, valproate ranked best for overall IGEs in efficacy and was the third best in tolerability. For the adjunctive therapies, topiramate ranked best for GTCA and overall IGEs, whereas levetiracetam ranked best for myoclonic seizures. Moreover, perampanel ranked best in tolerability measured by any TEAE when used either as a monotherapy or an adjunctive therapy. Overall, valproate is recommended as the monotherapy of choice for overall IGEs without contraindications. However, our results should be interpreted with caution considering the limited available information and the inherent methodological limitations of the NMA.

### Supplementary Information

Below is the link to the electronic supplementary material.

Supplementary file1 (DOCX 45 KB)

## Data Availability

All the datasets generated during the study are available on reasonable request from the corresponding author Xu Yang.
