# Supplementary material for: Antiseizure medications for idiopathic generalized epilepsies: a systematic review and network meta-analysis
Source: J Neurol. 2023 Jun 28;270(10):4713–28. doi: 10.1007/s00415-023-11834-8 (PMC10511599; doi:10.1007/s00415-023-11834-8)
Supplement: Supplementary file 1 — Supplementary file1 (DOCX 45 KB) [file 415_2023_11834_MOESM1_ESM.docx]

**Table S1 Search Strategy for involved studies**

**1.Absence seizure: Date：2022/12/17**

**PubMed database:**

| Term | No. | Search terms |
| --- | --- | --- |
| Epilepsy | 1 | absence[tiab] OR “absence seizure”[tiab] OR “childhood absence epilepsy”[tiab] OR “juvenile absence epilepsy”[tiab] OR “petit mal” [tiab] |
| Antiseizure medication | 2 | antiseizure medication[tiab] OR anti-epilep* [tiab] OR antiepileptic drugs [tiab] OR antiepileptic agents [tiab] OR anti-seizure*[tiab] OR anticonvulsant* [tiab] OR anti-epilep* [tw] OR anti-seizure*[tw] OR anticonvulsant* [tw] OR anticonvulsant* [Mesh] |
|  | 3 | Acetazolamide [tw] OR benzodiazepines [tw] OR brivaracetam [tw] OR carbamazepine [tw] OR clobazam [tw] OR clonazepam [tw] OR eslicarbazepine acetate [tw] OR ethosuximide [tw] OR felbamate [tw] OR gabapentin [tw] OR lacosamide [tw] OR lamotrigine [tw] OR levetiracetam [tw] OR nitrazepam [tw] OR oxcarbazepine [tw] OR perampanel [tw] OR phenobarbital [tw] OR phenytoin [tw] OR piracetam [tw] OR pregabalin [tw] OR primidone[tw] OR retigabine [tw] OR rufinamide [tw] OR stiripentol [tw] OR tiagabine [tw] OR topiramate [tw] valproic acid [tw] OR vigabatrin [tw] OR zonisamide [tw] |
| Type of study | 4 | randomized controlled trial [pt] OR controlled clinical trial [pt] OR randomized [tiab] OR placebo [tiab] OR clinical trials as topic [mesh: noexp] OR randomly [tiab] OR trial [ti]) NOT (animals [mh] NOT humans [mh] |
| Results by year | 1950-2022 | |
| Final strategy | 1AND (2 OR 3) AND 4 **354 results** | |

**Embase database:**

| Term | No. | Search terms |
| --- | --- | --- |
| Epilepsy | 1 | 'absence seizure'/exp OR 'petit mal'/exp OR (absence seizure*:ab,ti) OR (petit mal*:ab,ti) |
| Antiseizure medication | 2 | (anti-epilep*:ab,ti) OR (‘antiepileptic drugs’:ab,ti) OR (‘antiepileptic agents’:ab,ti) OR (anti-seizure*:ab,ti) OR (anticonvulsant*:ab,ti) OR anti-epilep*/exp OR anti-seizure*/exp OR anticonvulsant*/exp |
|  | 3 | ‘Acetazolamide’/exp OR ‘benzodiazepines’/exp OR ‘brivaracetam ’/exp OR ‘carbamazepine’/exp OR ‘clobazam’/exp OR ‘clonazepam’/exp OR ‘eslicarbazepine acetate’/exp OR ‘ethosuximide’/exp OR ‘felbamate’/exp OR ‘gabapentin’/exp OR ‘lacosamide’/exp OR ‘lamotrigine’/exp OR ‘levetiracetam’/exp OR ‘nitrazepam’/exp OR ‘oxcarbazepine’/exp OR ‘perampanel’/exp OR ‘phenobarbital’/exp OR ‘phenytoin’/exp OR ‘piracetam’/exp OR ‘pregabalin’/exp OR ‘primidone’/exp OR ‘retigabine’/exp OR ‘rufinamide’/exp OR ‘stiripentol’/exp OR ‘tiagabine’/exp OR ‘topiramate’/exp OR ‘vigabatrin’/exp OR ‘valproic acid’/exp OR ‘zonisamide’/exp |
| Type of study | 4 | 'crossover procedure':de OR 'double-blind procedure':de OR 'randomized controlled trial':de OR 'single-blind procedure':de OR (random* OR crossover* OR cross NEXT/1 over* OR placebo* OR doubl* NEAR/1 blind* OR singl* NEAR/1 blind* OR assign* OR allocat* OR volunteer*):de,ab,ti |
| Date limits | 5 | [1950-2022]/py |
| Final strategy | 1AND (2 OR 3) AND 4 AND 5 **995 results** | |

**Cochrance library database:**

| Term | No. | Search terms |
| --- | --- | --- |
| Epilepsy | 1 | (absence seizure OR petit mal OR absence seizure*):ti,ab,kw |
| Antiseizure medication | 2 | (anti-epilep* OR antiepileptic drugs OR antiepileptic agents OR anti-seizure* OR anticonvulsant*):ti,ab,kw |
|  | 3 | (Acetazolamide OR benzodiazepines OR brivaracetam OR carbamazepine OR clobazam OR clonazepam OR eslicarbazepine acetate OR ethosuximide OR felbamate OR gabapentin OR lacosamide OR lamotrigine OR levetiracetam OR nitrazepam OR oxcarbazepine OR perampanel OR phenobarbital OR phenytoin OR piracetam OR pregabalin OR primidone OR retigabine OR rufinamide OR stiripentol OR tiagabine OR topiramate OR vigabatrin OR valproic acid OR zonisamide):ti,ab,kw |
| Date limits | 4 | Between Jan 1950 and Nov 2022 |
| Final strategy | #1AND#2AND#3AND4 **10 results** | |

**2.Myoclonic seizure: Date：2022/1/29**

**PubMed database:**

| Term | No. | Search terms |
| --- | --- | --- |
| Epilepsy | 1 | myoclonic[tiab] OR “myoclonic seizure”[tiab] OR “juvenile myoclonic epilepsy”[tiab] |
| Antiseizure medication | 2 | antiseizure medication[tiab] OR anti-epilep* [tiab] OR antiepileptic drugs [tiab] OR antiepileptic agents [tiab] OR anti-seizure*[tiab] OR anticonvulsant* [tiab] OR anti-epilep* [tw] OR anti-seizure*[tw] OR anticonvulsant* [tw] OR anticonvulsant* [Mesh] |
|  | 3 | Acetazolamide [tw] OR benzodiazepines [tw] OR brivaracetam [tw] OR carbamazepine [tw] OR clobazam [tw] OR clonazepam [tw] OR eslicarbazepine acetate [tw] OR ethosuximide [tw] OR felbamate [tw] OR gabapentin [tw] OR lacosamide [tw] OR lamotrigine [tw] OR levetiracetam [tw] OR nitrazepam [tw] OR oxcarbazepine [tw] OR perampanel [tw] OR phenobarbital [tw] OR phenytoin [tw] OR piracetam [tw] OR pregabalin [tw] OR primidone[tw] OR retigabine [tw] OR rufinamide [tw] OR stiripentol [tw] OR tiagabine [tw] OR topiramate [tw] valproic acid [tw] OR vigabatrin [tw] OR zonisamide [tw] |
| Type of study | 4 | randomized controlled trial [pt] OR controlled clinical trial [pt] OR randomized [tiab] OR placebo [tiab] OR clinical trials as topic [mesh: noexp] OR randomly [tiab] OR trial [ti]) NOT (animals [mh] NOT humans [mh]) |
| Results by year | 1950-2022 | |
| Final strategy | 1AND (2 OR 3) AND 4 **58 results** | |

**Embase database:**

| Term | No. | Search terms |
| --- | --- | --- |
| Epilepsy | 1 | 'myoclonic seizure'/exp OR (myoclonic seizure*:ab,ti) |
| Antiseizure medication | 2 | (anti-epilep*:ab,ti) OR (‘antiepileptic drugs’:ab,ti) OR (‘antiepileptic agents’:ab,ti) OR (anti-seizure*:ab,ti) OR (anticonvulsant*:ab,ti) OR anti-epilep*/exp OR anti-seizure*/exp OR anticonvulsant*/exp |
|  | 3 | ‘Acetazolamide’/exp OR ‘benzodiazepines’/exp OR ‘brivaracetam ’/exp OR ‘carbamazepine’/exp OR ‘clobazam’/exp OR ‘clonazepam’/exp OR ‘eslicarbazepine acetate’/exp OR ‘ethosuximide’/exp OR ‘felbamate’/exp OR ‘gabapentin’/exp OR ‘lacosamide’/exp OR ‘lamotrigine’/exp OR ‘levetiracetam’/exp OR ‘nitrazepam’/exp OR ‘oxcarbazepine’/exp OR ‘perampanel’/exp OR ‘phenobarbital’/exp OR ‘phenytoin’/exp OR ‘piracetam’/exp OR ‘pregabalin’/exp OR ‘primidone’/exp OR ‘retigabine’/exp OR ‘rufinamide’/exp OR ‘stiripentol’/exp OR ‘tiagabine’/exp OR ‘topiramate’/exp OR ‘vigabatrin’/exp OR ‘valproic acid’/exp OR ‘zonisamide’/exp |
| Type of study | 4 | 'crossover procedure':de OR 'double-blind procedure':de OR 'randomized controlled trial':de OR 'single-blind procedure':de OR (random* OR crossover* OR cross NEXT/1 over* OR placebo* OR doubl* NEAR/1 blind* OR singl* NEAR/1 blind* OR assign* OR allocat* OR volunteer*):de,ab,ti |
| Date limits | 5 | [1950-2022]/py |
| Final strategy | 1AND (2 OR 3) AND 4 AND 5 **547 results** | |

**Cochrance library database:**

| Term | No. | Search terms |
| --- | --- | --- |
| Epilepsy | 1 | (myoclonic seizure OR myoclonic seizure*):ti,ab,kw |
| Antiseizure medication | 2 | (anti-epilep* OR antiepileptic drugs OR antiepileptic agents OR anti-seizure* OR anticonvulsant*):ti,ab,kw |
|  | 3 | (Acetazolamide OR benzodiazepines OR brivaracetam OR carbamazepine OR clobazam OR clonazepam OR eslicarbazepine acetate OR ethosuximide OR felbamate OR gabapentin OR lacosamide OR lamotrigine OR levetiracetam OR nitrazepam OR oxcarbazepine OR perampanel OR phenobarbital OR phenytoin OR piracetam OR pregabalin OR primidone OR retigabine OR rufinamide OR stiripentol OR tiagabine OR topiramate OR vigabatrin OR valproic acid OR zonisamide):ti,ab,kw |
| Date limits | 4 | Between Jan 1950 and Nov 2022 |
| Final strategy | #1AND#2AND#3AND4 **12 results** | |

**3.Idiopathic generalized epilepsy & Generalized tonic-clonic seizure alone**

**Pubmed database:**

| Term | No. | Search terms |
| --- | --- | --- |
| Epilepsy | 1 | generalized tonic-clonic seizure*[tiab] OR “idiopathic generalized epilepsy*”[tiab] OR “genetic generalized epilepsy*” [tiab] |
| Antiseizure medication | 2 | antiseizure medication[tiab] OR anti-epilep* [tiab] OR antiepileptic drugs [tiab] OR antiepileptic agents [tiab] OR anti-seizure*[tiab] OR anticonvulsant* [tiab] OR anti-epilep* [tw] OR anti-seizure*[tw] OR anticonvulsant* [tw] OR anticonvulsant* [Mesh] |
|  | 3 | Acetazolamide [tw] OR benzodiazepines [tw] OR brivaracetam [tw] OR carbamazepine [tw] OR clobazam [tw] OR clonazepam [tw] OR eslicarbazepine acetate [tw] OR ethosuximide [tw] OR felbamate [tw] OR gabapentin [tw] OR lacosamide [tw] OR lamotrigine [tw] OR levetiracetam [tw] OR nitrazepam [tw] OR oxcarbazepine [tw] OR perampanel [tw] OR phenobarbital [tw] OR phenytoin [tw] OR piracetam [tw] OR pregabalin [tw] OR primidone[tw] OR retigabine [tw] OR rufinamide [tw] OR stiripentol [tw] OR tiagabine [tw] OR topiramate [tw] valproic acid [tw] OR vigabatrin [tw] OR zonisamide [tw] |
| Type of study | 4 | randomized controlled trial [pt] OR controlled clinical trial [pt] OR randomized [tiab] OR placebo [tiab] OR clinical trials as topic [mesh: noexp] OR randomly [tiab] OR trial [ti]) NOT (animals [mh] NOT humans [mh]) |
| Results by year | 1950-2022 | |
| Final strategy | 1AND (2 OR 3) AND 4 **42results** | |

**Embase database:**

| Term | No. | Search terms |
| --- | --- | --- |
| Epilepsy | 1 | (idiopathic generalized epilepsy*:ab,ti) OR (generalized tonic-clonic seizure*:ab,ti) |
| Antiseizure medication | 2 | (anti-epilep*:ab,ti) OR (‘antiepileptic drugs’:ab,ti) OR (‘antiepileptic agents’:ab,ti) OR (anti-seizure*:ab,ti) OR (anticonvulsant*:ab,ti) OR anti-epilep*/exp OR anti-seizure*/exp OR anticonvulsant*/exp |
|  | 3 | ‘Acetazolamide’/exp OR ‘benzodiazepines’/exp OR ‘brivaracetam ’/exp OR ‘carbamazepine’/exp OR ‘clobazam’/exp OR ‘clonazepam’/exp OR ‘eslicarbazepine acetate’/exp OR ‘ethosuximide’/exp OR ‘felbamate’/exp OR ‘gabapentin’/exp OR ‘lacosamide’/exp OR ‘lamotrigine’/exp OR ‘levetiracetam’/exp OR ‘nitrazepam’/exp OR ‘oxcarbazepine’/exp OR ‘perampanel’/exp OR ‘phenobarbital’/exp OR ‘phenytoin’/exp OR ‘piracetam’/exp OR ‘pregabalin’/exp OR ‘primidone’/exp OR ‘retigabine’/exp OR ‘rufinamide’/exp OR ‘stiripentol’/exp OR ‘tiagabine’/exp OR ‘topiramate’/exp OR ‘vigabatrin’/exp OR ‘valproic acid’/exp OR ‘zonisamide’/exp |
| Type of study | 4 | 'crossover procedure':de OR 'double-blind procedure':de OR 'randomized controlled trial':de OR 'single-blind procedure':de OR (random* OR crossover* OR cross NEXT/1 over* OR placebo* OR doubl* NEAR/1 blind* OR singl* NEAR/1 blind* OR assign* OR allocat* OR volunteer*):de,ab,ti |
| Date limits | 5 | [1950-2022]/py |
| Final strategy | 1AND (2 OR 3) AND 4 AND 5 **764results** | |

**Cochrance library database:**

| Term | No. | Search terms |
| --- | --- | --- |
| Epilepsy | 1 | (idiopathic generalized epilepsy* OR generalized tonic-clonic seizure*):ti,ab,kw |
| Antiseizure medication | 2 | (anti-epilep* OR antiepileptic drugs OR antiepileptic agents OR anti-seizure* OR anticonvulsant*):ti,ab,kw |
|  | 3 | (Acetazolamide OR benzodiazepines OR brivaracetam OR carbamazepine OR clobazam OR clonazepam OR eslicarbazepine acetate OR ethosuximide OR felbamate OR gabapentin OR lacosamide OR lamotrigine OR levetiracetam OR nitrazepam OR oxcarbazepine OR perampanel OR phenobarbital OR phenytoin OR piracetam OR pregabalin OR primidone OR retigabine OR rufinamide OR stiripentol OR tiagabine OR topiramate OR vigabatrin OR valproic acid OR zonisamide):ti,ab,kw |
| Date limits | 4 | Between Jan 1950 and Nov 2022 |
| Final strategy | #1AND#2AND#3AND4 **8 results** | |

**Table S2. RoB 2 Assessment for Risk of Bias**

| Study ID | Over all Bias | Randomization Process | Deviations from intended intervention | Missing outcom data | Measurement of the Outcome | Selection of the Repoted Result |
| --- | --- | --- | --- | --- | --- | --- |
| AE | | | | | | |
| Basu 2005 | high risk | some concerns | some concerns | high risk | low risk | some concerns |
| Callaghan 1982 | Low risk | some concerns | low risk | low risk | low risk | low risk |
| Cnaan 2017 | Low risk | Low risk | low risk | low risk | low risk | Low risk |
| Coppola 2004 | Low risk | Low risk | low risk | low risk | low risk | Low risk |
| Fattore 2011 | Low risk | Low risk | low risk | low risk | low risk | Low risk |
| Frank 1999 | Low risk | Low risk | low risk | low risk | low risk | Low risk |
| Glauser 2010&2013 | Low risk | Low risk | low risk | low risk | low risk | Low risk |
| Huang 2009 | Low risk | Low risk | low risk | low risk | low risk | Low risk |
| Shinnar 2017 | Low risk | Low risk | low risk | low risk | low risk | Low risk |
| Hwang 2011 | high risk | high risk | Low risk | high risk | some concerns | Low risk |
| ME | | | | | | |
| Machado 2013 | high risk | high risk | low risk | low risk | low risk | Low risk |
| Nejad 2009 | high risk | high risk | low risk | high risk | some concerns | high risk |
| Noachtar 2008 | low risk | low risk | low risk | low risk | low risk | Low risk |
| Levisohn 2007 | high risk | high risk | low risk | low risk | low risk | Low risk |
| IGEs | | | | | | |
| SANDA arm B 2007 | high risk | high risk | low risk | low risk | low risk | Low risk |
| SANDA II arm B 2021 | high risk | high risk | low risk | low risk | low risk | some concerns |
| Berkovic 2007 | Low risk | Low risk | low risk | low risk | low risk | Low risk |
| Brandt 2020 | Low risk | Low risk | low risk | low risk | low risk | Low risk |
| GTCA | | | | | | |
| Driscoll 2020 | Low risk | Low risk | low risk | low risk | low risk | Low risk |
| French 2015 | Low risk | Low risk | low risk | low risk | low risk | Low risk |
| French 2020 | Low risk | Low risk | low risk | low risk | low risk | Low risk |
| Prakash 2016 | high risk | high risk | some concerns | low risk | low risk | low risk |
| Vossler 2020 | Low risk | Low risk | low risk | low risk | low risk | Low risk |
| Wu 2018 | Low risk | Low risk | low risk | low risk | low risk | Low risk |
| Biton 2010 | Low risk | Low risk | low risk | low risk | low risk | Low risk |
| Beran 1998 | some concerns | some concerns | some concerns | low risk | low risk | Low risk |
| Biton 1999 | Low risk | Low risk | low risk | low risk | low risk | Low risk |

AE: absence epilepsy, ME: myoclonic epilepsy, IGEs: idiopathic generalized epilepsies, GTCA: generalized tonic-clonic seizures alone. “/” represents not mentioned or not taken down for reasons.

**Table S3. Ranking sequence according to SUCRA for the efficacy and tolerability outcomes of treatments**

1. Seizure free of 3-6 month after monotherapy in overall IGEs

| Treatment | ESM | VPA | TPM | Placebo | LTG |
| --- | --- | --- | --- | --- | --- |
| SUCRA | 0.833 | 0.658 | 0.594 | 0.228 | 0.186 |

B) Seizure free of 3-6 month after adjunctive therapy in overall IGEs

| Treatment | adTPM | adLEV | adLCM | adPER | adLTG | adPlacebo |
| --- | --- | --- | --- | --- | --- | --- |
| SUCRA | 0.890 | 0.830 | 0.5 | 0.413 | 0.279 | 0.087 |

C)Seizure free of 12-month after monotherapy in overall IGEs (long term follow-up)

| Treatment | ESM | VPA | TPM | LEV | LTG |
| --- | --- | --- | --- | --- | --- |
| SUCRA | 0.891 | 0.660 | 0.450 | 0.438 | 0.006 |

D)Seizure free of 3-6 month after monotherapy in AE

| Treatment | ESM | VPA | Placebo | LTG |
| --- | --- | --- | --- | --- |
| SUCRA | 0.903 | 0.686 | 0.233 | 0.178 |

E) Seizure free of 3-6 month after adjunctive therapy in ME

| Treatment | adLEV | adLTG | adPER | adPlacebo |
| --- | --- | --- | --- | --- |
| SUCRA | 0.888 | 0.433 | 0.417 | 0.262 |

F) Seizure free of 3-6 month after adjunctive therapy in GTCA

| Treatment | adTPM | adLCM | adPER | adLTG | adPlacebo |
| --- | --- | --- | --- | --- | --- |
| SUCRA | 0.929 | 0.575 | 0.494 | 0.361 | 0.141 |

G) Any adverse event after adjunctive therapy in overall IGEs

| Treatment | adPlacebo | adLEV | adPER | adLTG | adLCM |
| --- | --- | --- | --- | --- | --- |
| SUCRA | 0.832 | 0.649 | 0.534 | 0.098 | 0.387 |

H) Any adverse event after monotherapy in overall IGEs

| Treatment | PER | Placebo | LTG | VPA | LEV | ESM | TPM |
| --- | --- | --- | --- | --- | --- | --- | --- |
| SUCRA | 0.837 | 0.728 | 0.532 | 0.506 | 0.387 | 0.313 | 0.198 |

I) Adverse event leading to discontinue after adjunctive therapy in overall IGEs

| Treatment | adPlacebo | adPER | adTPM | adLEV | adLCM | adLTG |
| --- | --- | --- | --- | --- | --- | --- |
| SUCRA | 0.698 | 0.656 | 0.607 | 0.510 | 0.362 | 0.167 |

J) Adverse event leading to discontinue after monotherapy in overall IGEs (part 1)

| Treatment | VPA | TPM | ESM | LTG |
| --- | --- | --- | --- | --- |
| SUCRA | 0.641 | 0.559 | 0.417 | 0.384 |

K) Adverse event leading to discontinue after monotherapy in overall IGEs (part 2)

| Treatment | Placebo | LEV | PER |
| --- | --- | --- | --- |
| SUCRA | 0.980 | 0.484 | 0.036 |

SUCRA=surface under the cumulative ranking curve. Higher SUCRA values correspond to higher probabilities of better efficacy/tolerability. AE: absence epilepsy, ME: myoclonic epilepsy, IGEs: idiopathic generalized epilepsies, GTCA: generalized tonic-clonic seizures alone. VPA: valproate, LTG: lamotrigine, TPM: topiramate, LEV: levetiracetam, ESM: ethosuximide, PER: perampanel, LCM: lacosamide, ad : adjunctive
